# Interventions for Improving Nutrition and Physical Activity Behaviors in Adult African American Populations: A Systematic Review, January 2000 Through December 2011

**DOI:** 10.5888/pcd10.120256

**Published:** 2013-06-20

**Authors:** Jennifer Lemacks, Brittny A. Wells, Jasminka Z. Ilich, Penny A. Ralston

**Affiliations:** Author Affiliations: Brittny A. Wells, Florida Agricultural and Mechanical University, Tallahassee, Florida; Jasminka Z. Ilich, Penny A. Ralston, Florida State University, Gainesville, Florida.

## Abstract

**Introduction:**

The incidence of preventable chronic diseases is disproportionally high among African Americans and could be reduced through diet and physical activity interventions. Our objective was to systematically review the literature on clinical outcomes of diet and physical activity interventions conducted among adult African American populations in the United States.

**Methods:**

We used the Preferred Reporting Items for Systematic Review and Meta Analysis construct in our review. We searched Medline (PubMed and Ovid), Cochrane, and DARE databases and restricted our search to articles published in English from January 2000 through December 2011. We included studies of educational interventions with clinically relevant outcomes and excluded studies that dealt with nonadult populations or populations with pre-existing catabolic or other complicated disorders, that did not focus on African Americans, that provided no quantitative baseline or follow-up data, or that included no diet or physical activity education or intervention. We report retention and attendance rates, study setting, program sustainability, behavior theory, and education components.

**Results:**

Nineteen studies were eligible for closer analysis. These studies described interventions for improving diet or physical activity as indicators of health promotion and disease prevention and that reported significant improvement in clinical outcomes.

**Conclusion:**

Our review suggests that nutrition and physical activity educational interventions can be successful in improving clinically relevant outcomes among African Americans in the United States. Further research is needed to study the cost and sustainability of lifestyle interventions. Further studies should also include serum biochemical parameters to substantiate more specifically the effect of interventions on preventing chronic disease and reducing its incidence and prevalence.

## Introduction

Education and community-based programs in disease prevention and health promotion played an important role in achieving *Healthy People 2010* (1) goals and are of continued importance in attaining *Healthy People 2020* goals, especially among minority populations (2). Primary prevention interventions aimed at reducing the higher incidence and prevalence of chronic diseases among African Americans (3–5) include education about healthful lifestyle choices regarding diet and physical activity. Interest in and funding for research into the health effects of improved nutrition and physical activity is needed to develop interventions that would reduce disparities between African Americans and the US population overall.

Growing awareness of the role of a healthful diet and physical activity in reducing chronic disease has greatly increased the volume of recent literature on the subject, so that many reviews (6–9) are now outdated. Researchers have recommended using more rigorous methodology (6,9) and have discussed validated and reliable instruments (6,9), theory-based interventions (9), culturally tailored interventions (6,7), and concepts for successful recruitment and retention of diverse study populations (8). Researchers have not fully addressed clinical outcomes. For example, a recent review (10) examined physical activity outcomes (ie, increasing frequency of exercise) among African Americans but did not evaluate various health-related outcomes (ie, weight loss or improved lipid profile) resulting from increased exercise. Few reviews focused on clinical outcomes from improved diet and nutrition (6–10). Our objective was to review available literature on the effect of educational interventions on clinical outcomes resulting from improved nutrition and increased physical activity among adult African Americans in the United States.

## Methods

### Data sources

We used the Preferred Reporting Items for Systematic Reviews and Meta Analysis (PRISMA) (11) for this review. We searched Medline (PubMed and Ovid) and Cochrane databases and DARE (Database of Abstracts of Reviews of Effects) to identify effective interventions in modifying dietary and physical activity behaviors among adult African Americans in the United States, January 2000 through December 2011.

### Study selection

Nineteen studies (12–32) met our selection criteria, were confirmed by 2 reviewers, and were included in this review (Figure); 1 study reported dietary and physical activity components of the intervention in 2 separate articles (20,21), and another reported intervention results in 1 publication (26) and recruitment and retention information in another (27). We restricted our search to articles published in English from January 1, 2000, through December 31, 2011. Articles were limited to the past 12 years to capture the most recent studies; earlier studies had been included in previous reviews (6–9). Research and publishing on the effect of diet and physical activity interventions among minority and underserved populations have increased during the past 12 years in the United States, providing adequate literature for review.

**Figure:**
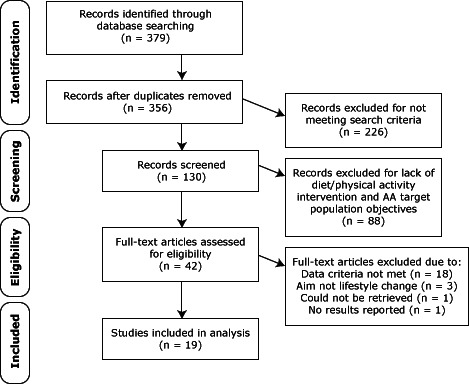
Data-filtering process used to select final 19 studies included in systematic review of interventions for improving nutrition and physical activity behaviors among adult African American populations, January 2000 through December 2011. Flow diagram for study selections adapted from PRISMA (11).

### Data extraction

Our initial search used a combination of the following key words: African American, nutrition, diet, physical activity, weight loss, and intervention. The inclusion criteria were 1) that the article addressed educational interventions in diet and physical activity with clinically relevant outcomes (eg, changes in weight, body mass index (BMI [kg/m^2^]), body fat percentage, cholesterol, triglycerides, blood pressure) and 2) that the educational interventions included at least 1 direct or indirect instructional strategy to educate participants on how to increase daily physical activity and fruit and vegetable consumption or how inactivity and poor dietary patterns can negatively affect health. We excluded studies that dealt with nonadult populations or populations with pre-existing catabolic or other complicated disorders (eg, cancer, cirrhosis, HIV/AIDs, heart failure); studies that did not focus on African Americans; studies that provided no quantitative baseline or follow-up data; and studies that included no diet or physical activity education or intervention. Data were extracted by 1 reviewer and verified by a second. Information on the following components were collected: study settings, theoretical principles incorporated into the intervention design, study outcomes, intervention details, recruitment strategies, attendance and retention rates of the participants, sustainability of the achieved changes in health behaviors, and provision of physical activity and nutrition education.

## Results

### Characteristics of analyzed studies

Among the 19 studies reviewed (5.3% of nonduplicated identified articles), 1 included participants of various races and ethnicities (13), and 4 (12,14,15,32) included both white and African American participants; however, all 4 had sizable samples and distinguished the African American population (according to the US Census Bureau 2002, 13% of the total US population are African Americans [33]) (Table). Mean age of participants in all studies (N = 3,530) was 51.5 years; 2,997 (85.0%) were women and 533 (15.0%) were men. One study (18) did not report mean age of participants but enrolled only 10 participants. Another study (13) was not represented in the mean age because it reported age data in ranges. The majority of men (63.5%) and women (66.9%) were aged between 40 and 50 years. A study by Goodpaster et al (14) with 48 participants was not represented in the mean sample evaluation because it was not possible to distinguish African American men and women from the total sample number. At baseline, mean BMI was 34.7 (n = 2,613; range, 30.3–38.9), mean systolic blood pressure (SBP) was 135.1 mm Hg (n = 1,868; range, 120.8–151.4), and mean diastolic blood pressure (DBP) was 81.1 mm Hg (n = 1,868; range, 74.0–91.0) (17–21,23–29,31,32). We excluded 2 studies (12,14) from these calculations for not distinguishing African American population characteristics; 2 studies (13,16) for not including BMI information, and 3 studies (15,22,30) for not including blood pressure information.

**Table:** Randomized and Nonrandomized Diet and Physical Activity Intervention Studies in Adult African American Populations, January 2000 Through December 2011

Study Components	Description
**Randomized Controlled Trials, Significant Results Reported**	
**West et al, 2008 (** 13 **)**	
Sample	N = 508 African Americans (341 women, 167 men) aged ≥18 years with type 2 diabetes. Unable to determine mean age and standard deviation [SD] for participants because of inability to distinguish African American participants from other races. Three groups: metformin group, 163 women and 58 men who received standard lifestyle recommendations plus metformin; placebo group, 163 women and 57 men who received standard lifestyle recommendations plus placebo; lifestyle group, 154 women and 50 men who received intensive lifestyle modification.
Theory	None noted
Study outcome(s)	Weight
Intervention	Duration: 6 months. Location: various clinical centers. Design: individual or one-on-one sessions; consisted of 16 diet and lifestyle sessions using the NIH DPP over 4 months from initiation of intervention with two additional monthly follow up sessions. Education: diet and PA. Follow-up: at 6 months, 12 months, 18 months, 24 months, and 30 months.
Attendance and retention	Attendance: not reported. Retention: 91% women, 87% men.
Clinical outcomes and results	Weight loss: 4.8 kg, women, 2.1 kg, men. Results significant over 30 month (*P* < .05) in intensive lifestyle modification group with no significant difference between placebo or metformin groups. Results reported exclusive to the African American subgroup of study.
**Goodpaster, et al, 2010 (** **14** **)**	
Sample	48 African American women and men aged 30–55 y (mean 46.8 y, SD, 6.4 y), BMI ≥35 kg/m^2^. Two groups: initial PA, delayed PA.
Theory	None noted
Study outcome	Weight, waist circumference. abdominal adiposity, visceral fat, body composition (DXA measure), BP.
Intervention	Duration: 12 months. Location: university. Design: group, individual, and telephone contacts (1st–6th month, 3 group and 1 individual contacts/month; 6th–12th month, 2 group sessions and 2 telephone contacts/month). Diet prescribed for both groups to achieve 8% to 10% weight loss over 12 months; meal replacements offered during first 6 months. PA goals for both groups to achieve 60 minutes, 5 days per week, moderate-intensity PA. Delayed PA group started PA at 6th month. Education: diet and physical activity. Follow-up: 6 months, and 12 months.
Attendance and retention	Attendance: not reported. Retention: initial group, 90% at 6 months, 73% at 12 months; delayed group, 90% at 6 months, 83% at 12 months. Study not African American- specific; however, no difference reported between white and African American participants.
Clinical outcomes and results	Weight: 12.1 kg decrease for initial group; 9.9 kg decrease for delayed group (*P* < .001). Loss in percentage body fat and waist circumference significant in both groups (*P* < .001); higher in initial group but not significant. SBP: approximately 15 mm Hg decrease at 12 months in initial and delayed groups (*P* < .001). DBP: approximately 6 mm Hg decrease in initial and delayed groups (*P* < .001). Insulin and HOMA-IR: significant decrease (insulin, approximately 4µU/mL; HOMA-IR, approximately 1.0 point, [*P* ≤ .01]); FG, total cholesterol, HDL, triglycerides: no significant differences. Study not African American specific; however, reported no difference between white and African American groups. Results based on 12–month follow up results compared with 0 month; trends similar at 6th month with exception of no significant change in BP.
**Murrock et al, 2009 (** **6** **)**	
Sample	Women (N = 70) with type 2 diabetes aged ≥18 years (mean, 62.8 y; SD, 10.1 y). Two groups: group 1 (n = 34), dance intervention plus usual care; group 2 (control group) (n = 36), usual care.
Theory	Social cognitive learning theory
Study outcome(s)	HbA1c, weight, BIA, BP
Intervention	Duration: 12 weeks. Location, community center. Design, group, 60-minute dance intervention 2 times per week, led by experienced African American instructor. Education: diet and physical activity. Follow up: 12th week.
Attendance, retention	Attendance: not reported. Retention: 84%.
Clinical outcomes and results	HbA1c: decrease 0.5% in dance group (*P* < .05); increase 0.3% in control group (*P* < .05). Weight: decrease 10.8 kg in dance group (*P* < .01); increase 8.1 kg in control group (*P* < .01). Percentage body fat (BIA measure): decrease 2.8% in dance group (*P* < .01); no change in control group. SBP: 8.8 mm Hg decrease (*P* < .01) in dance group; 4.1 mm Hg increase in control group. DBP: 10.2 mm Hg decrease (*P* < .05) in dance group; 1.4 mm Hg decrease in control group. All results compared with baseline.
**Yanek et al, 2001 (** **17** **)**	
Sample	Women (n = 529) ≥40 y (mean, 53.1 y; SD, 9.3 y). Three groups: group without spirituality (n = 188); group 2, group with spirituality (n = 267); group 3, self-help group, no spirituality (n = 74).
Theory	None noted
Study outcome(s)	Weight, BMI, waist circumference, BP, blood lipids, FG, energy intake, smoking, PA
Intervention	Duration: 20 weeks. Location: 16 churches. Design: weekly group meetings of 30 to 45 minutes with nutrition education and 30 minutes moderate aerobic exercise. First 20 weeks, group led by research staff; thereafter, by church lay health leader. Education: diet and PA. Follow-up: 6 months, 12th months.
Attendance and retention	Attendance, 33%–50% overall. Retention: 71.2%.
Clinical outcomes and results	Weight: 0.5 kg decrease in without-spirituality and spirituality groups (*P* ≤ .001); 0.8 kg increase in self-help group (*P* < .01). BMI, 0.2 kg/m^2^ decrease in without-spirituality and spirituality groups (*P* = .02); 0.1kg/m^2^ increase in self-help group (not significant). Waist circumference: 1.7 cm decrease in without-spirituality and spirituality groups (*P* < .001); 0.02 cm decrease in self-help group (not significant). SBP: 1.6 mm Hg decrease in without-spirituality and spirituality groups (*P* < .001); 0.9 mm Hg decrease in self-help group (no significance). DBP: 0.4 mm Hg decrease in without-spirituality and spirituality groups; 0.2 mm Hg increase in self-help group. Results based on 12th month follow up.
**Kennedy et al, 2005 (** **19** **)**	
Sample	Overweight or obese women (n = 37) and men (n = 3), ≥20 y of age (mean, 44.0 y, SD, 10.0 y). Two groups: group intervention (n = 20), individual intervention (n = 20).
Theory	None noted
Study outcome(s)	PA, weight, body composition (DXA measure), blood lipids, FG, BP, quality of life
Intervention	Duration: 6 months. Location: church. Design: group and individual (or one-one-one). Intervention conducted by lay health educators with extensive training. Group intervention had 6 monthly meetings; individual intervention had 15 meetings over 6 months. Education: diet and PA. Follow-up: 6th month.
Attendance and retention	Attendance: not reported. Retention: 90%.
Clinical outcomes and results	Weight: mean loss 3.3 kg in both groups (*P* < .05); no significance between groups. BMI: 1.2 decrease in both groups; no significance compared with baseline or between groups. Percentage body fat: mean loss 0.5% in both groups (*P* < .05); no significance between groups. SBP: 1 mm Hg loss in both groups, no significance compared with baseline or between groups. DBP: 2 mm Hg loss in both groups; no significance compared with baseline or between groups. HDL, 3 mg/dL decrease. LDL: 4 mg/dL decrease (*P* < .05 at baseline); no significance between groups. Total cholesterol, triglycerides, FG: no significant differences from baseline or between groups.
**Ard et al, 2000 (** **24** **)**	
Sample	N = 56 (54 women, 2 men). Mean age, 40.4 y; SD, not reported. Two groups: intervention (n = 35); delayed (n = 22).
Theory	None noted
Study outcome(s)	Weight, BP, cholesterol
Intervention	Duration: 8 weeks. Location: university. Design: both groups led by African American instructor; both groups prescribed progressive diet (1st week, rice diet, 1,000 kcal, 7% fat; 3rd week, added animal protein (eggs, milk, cheese); 5th week, advanced animal protein (lean meat [chicken/fish], 1,200 kcal, 14% fat). Delayed group began intervention after 8 weeks. Participants paid $106 for university employees and $170/nonemployees to cover food costs. Education: diet and physical activity. Follow-up: 8 weeks.
Attendance and retention	Attendance: 79%. Retention: 77%.
Clinical outcomes and results	Weight: 32.6 kg mean loss (*P* < .01). BMI: 2.5 mean decrease (*P* < .01). SBP: 4.3 mm Hg mean decrease (*P* < .01). DBP: 2.5 mm Hg mean decrease (*P* < 0.05). Cholesterol: 13.7 mg/dL decrease (*P* < .01).
**Staffileno et al, 2007 (** **26** **); Staffileno and Coke, 2006 (** **27** **)^a^**	
Sample	N = 24 women, aged 18 to 45 (mean, 39 y; SD, 5.5 y), sedentary, with normal BP or stage 1 hypertension. Two groups: group 1 (n = 13), exercise; group 2 (n = 10), no exercise.
Theory	None noted
Study outcome(s)	BP, PA
Intervention	Duration: 8 weeks. Location: participant’s home. Design: individual. Exercise group visited at home to encourage lifestyle PA (eg, walking, stair climbing) for 10 minutes, 3 times a day, 5 days a week following NIH-DPP program. Education: PA only. Follow-up: 8th week.
Attendance and retention	Attendance: not reported. Retention: 96%.
Clinical outcomes and results	SBP: 6.4 mm Hg decrease (*P* < .05) in exercise group; significantly different from no-exercise group (*P* = .04). DBP: 3.4 mm Hg decrease in exercise group, not significantly different from no-exercise group.
**Wilbur et al, 2008 (** **29** **)**	
Sample	N = 281 women, aged 40 to 65 y (mean, 48.6 y; SD, 6.0 y). Two groups: intervention (n = 156), control (n = 125).
Theory	None noted
Study outcome(s)	BMI, waist circumference
Intervention	Duration: 48 weeks. Location: community health centers. Design: intervention group, 4 targeted workshops followed by weekly telephone calls over 24 weeks. Education: PA only. Follow-up: week 24 and week 48.
Attendance and retention	Attendance: 58%, intervention; 25%, control. Retention: 42.7%.
Clinical outcomes and results	BMI: 0.7 decrease in intervention group; 0.2 decrease in control group; no significant difference between groups. Waist circumference: 1.1 cm decrease in intervention group (*P* < .05); 1.1 cm decrease in control group; not significant. Results based on 24th week and maintained at 48th week.
**Samuel-Hodge et al, 2009 (** **31** **)**	
Sample	N = 201 (128 women, 73 men) aged ≥20 y (mean, 59.2 y; SD, 1.1 y) with diabetes diagnosis. Two groups: special intervention (n = 117 [75 women, 17 men]); minimal intervention (n = 84 [53 women, 31 men]).
Theory	None noted
Study outcome(s)	Diet, PA, diabetes self-management
Intervention	Duration: 48 weeks. Location: community health centers. Design: special intervention group, 8-month intensive phase with 1 counseling visit, 12 group sessions, telephone calls, 3 postcards, and 4-month reinforcement phase with telephone calls; minimal intervention group, standard education pamphlets by mail. Education: diet and PA. Follow-up: 8th and 12th months.
Attendance and retention	Attendance: 67% special intervention group and 70%, minimal intervention group at 8th month; 68% special intervention group, 67% minimal intervention group at 12th month. Retention: 84.5%.
Clinical outcomes and results	HbA1c: 0.4% decrease in special intervention; no decrease in minimal intervention (*P* < .01). BP: nonsignificant change in both groups. DBP: 3.3 mm Hg lower in minimal intervention group than in special intervention group (*P* < .001). Results based on 8-month follow-up and were attenuated at 12 months.
**Mayer-David et al, 2004 (** **32** **)**	
Sample	N = 152 (123 women, 29 men with diabetes). Mean age, 60.3 y; SD, 8.6 y. Three groups: intensive-lifestyle intervention (n = 49); reimbursable-lifestyle intervention (n = 47); control (usual care) (n = 56).
Theory	None noted
Study outcome(s)	Weight
Intervention	Duration: 12 months. Location: community health centers. Design: intensive lifestyle intervention group met weekly with nutritionist for first 4 months, every other week for next 2 months, once monthly for remaining 6 months; reimbursable lifestyle intervention group had key elements of intensive lifestyle intervention group delivered in four 1-hour sessions (equivalent to the amount of allowable time to reimbursed for nutrition counseling by Medicaid) over 12 months with 3 group sessions and 1 individual session; program modeled after NIH DPP. Education: diet and PA. Follow-up: 3rd, 6th, and 12th months.
Attendance and retention	Attendance: not reported. Retention: 81%.
Clinical outcomes and results	Weight: 2.2 kg loss in intensive lifestyle intervention group; significant compared with baseline (*P* < .05) and control (*P* = .05). No significant difference between reimbursable lifestyle intervention group and control group. BMI: 0.97 decrease in intensive lifestyle intervention group; 0.16 decrease in control group; significant from baseline (*P* < .001) and control (*P* < .01). HbA1c, triglycerides, HDL, LDL, SBP, DBP: no significant difference between reimbursable lifestyle intervention, intensive lifestyle intervention, and control. Results based on 12th month.
**Fitzgibbon et al, 2005 (** **30** **)**	
Sample	N = 59 overweight/obese women. Mean age 48.5 y; SD, 21.9 y. Two groups: faith-based weight-loss intervention (n = 30), weight-loss intervention (n = 29).
Theory	SCT
Study outcome(s)	Weight, dietary fat consumption, PA
Intervention	Duration: 12 weeks. Location: hospital. Design: Groups met 2 times per week; weight-loss group received culturally tailored intervention; faith-based group received same intervention with addition of a faith/spirituality component. Education: diet and PA. Follow-up: 12th week.
Attendance and retention	Attendance: 54% faith-based weight-loss group; 54%, weight-loss group. Retention: 77%, faith-based weight-loss group; 79% weight-loss group.
Clinical outcomes and results	Weight loss: 2.6 kg, faith-based weight-loss group (*P* < .01); 1.6 kg, weight-loss group (*P* < .05). BMI: 1.0 decrease in faith-based weightloss group (*P* < .01); 0.6 kg decrease in weight-loss group (*P* < .05). Results compared with baseline; no significant differences between treatment groups.
**Zoellner et al, 2011 (** **12** **)**	
Sample	N = 269 (229 women, 40 men). Mean age, 43.8 y; SD, 12.1 y.
Theory	Community-based participatory research, transtheoretical model
Study outcome(s)	BP, PA
Intervention	Duration: 18 months. Location: community. Design: trained and paid community coach-led groups participated in intervention with monthly 90-minute group education and PA sessions; used pedometers and diaries. Education: PA only. Follow-up: 3rd, 6th, 12th, and 18th months.
Attendance and retention	Attendance: 33.6%. Retention: 84%.
Clinical outcomes and results	SBP: 6 mm Hg decrease (*P* < .001). DBP: 3 mm Hg decrease (*P* < .001). Results based on 3-month follow-up (no other follow-up currently available) and compared with baseline.
**Duru et al., 2010 (** **28** **)**	
Sample	N = 71 women aged ≥60 years (mean, 72.8 y; SD, 7.7 y). Two groups: intervention (n = 37); control (n = 34).
Theory	None noted
Study outcome(s)	Weight, BP, PA, chronic pain
Intervention	Duration: 6 months. Location: church. Design: Both groups received 45 minutes PA per week for 8 weeks and then once monthly for 4 months. Intervention group had additional 45 minutes PA faith-based curriculum; both groups used pedometer. Education: diet and PA. Follow-up: 6th month.
Attendance and retention	Attendance: 85% (75% of classes). Retention: 87%.
Clinical outcomes and results	Weight: 1.0 kg decrease, intervention group; 0.7 kg decrease, control group; no significant difference between groups. BMI: not reported. SBP: 12.5 mm Hg decrease, intervention group; 1.5 mm Hg decrease control group (*P* < .01). DBP: 5.9 mm Hg decrease, intervention group; 3.8 mm Hg decrease, control group; no significant difference between groups.
**Randomized Trials, Nonsignificant Results Reported**	
**McCarthy et al, 2007^a^ (** **20** **), Yancey et al, 2006^b^ (** **21** **)**	
Sample	N = 366 women. Mean age, 45.5 y; SD, 10.5 y. Two groups: intervention group: (n = 188); control group, (n = 178).
Theory	SCT, socio-ecological model
Study outcome(s)	Diet quality (fiber, fruits and vegetables), body fat percentage (BIA measure), waist circumference, fitness
Intervention	Duration: 8 weeks. Location: local gym. Design: group; 1 hour PA, 1 hour nutrition lectures and activities; participants given 1 year free gym membership; administered food frequency questionnaire; recorded 1 mile run/walk; waist circumference, percentage body fat (BIA measure). Education: diet and PA. Follow-up: 2nd, 6th, 12th month.
Attendance and retention	Attendance: 80%–95% 1 or more sessions. Retention: >70%.
Clinical outcomes and results	BMI: marginal decrease (*P* = .06) with no sustained results
**Nonrandomized Trials, Significant Results Reported**	
**Stolley et al, 2009 (** **22** **)**	
Sample	N = 20 overweight women. Mean age, 51.4 years; SD, 8.9 y.
Theory	SCT, health behavior theory
Study outcome(s)	Social support, food intake, weight, BMI, PA
Intervention	Duration: 6 months. Location: community center. Design: 2 weekly group classes: 1st hour, education; 2nd hour, exercise. Education: diet and PA. Follow-up: 6th month.
Attendance and retention	Attendance: 55% (75% of classes). Retention: 87%.
Clinical outcomes and results	Weight: 14.5 kg loss (*P* = .001). BMI: 1.0 decrease (*P* = .001).
**Boltri et al, 2008 (** **25** **)**	
Sample	N = 46 (26 women, 20 men) aged ≥18 years at risk for diabetes. Mean age, 52.1 y; SD not reported.
Theory	None noted
Study outcome(s)	Weight, BP, FG
Intervention	Duration: 4 months. Location: church. Design: individual; 16 NIH DPP sessions. Education: diet and PA. Follow-up: 6th, 12th month.
Attendance and retention	Attendance: not reported. Retention: 65%.
Clinical outcomes and results	Weight: 2.5 kg loss at 6 months, significant compared with baseline (*P* < .05). BMI: 0.9 decrease at 6 months, significant compared with baseline (*P* < .05). SBP: 9 mm Hg decrease; DBP, 6 mm Hg decrease; FG, 4.0 mg/dL decrease at 6 months compared with baseline (*P* < .05). Results maintained significance at 12 months; SBP, DBP, FG improved.
**Hollis et al, 2008 (** **15** **)**	
Sample	N = 736 African American (540 women, 196 men) aged ≥ 25 years (mean, 52.3 y; SD, 9.5 y); BMI 25–45 kg/m^2^. Phase I trial.
Theory	SCT, transtheoretical model
Study outcome(s)	Weight, PA
Intervention	Duration: 20 weeks. Location: 4 clinical research centers. Design: group conducted by nutrition and behavioral counselors with goals of achieving ≥4 kg weight loss and 180 minutes per week moderate-intensity PA (need to enter Phase II trial). Education: diet and PA. Follow-up: 0, 20 weeks (participants achieving goals were entered into Phase II).
Attendance and retention	Attendance: 71% (not African American specific). Retention: 92% (not African American specific).
Clinical outcomes and results	Weight: men, 5.4 kg mean loss; women, 4.1 kg mean loss (*P* < .01). BMI: men, 1.7 decrease; women, 1.4 decrease.
**Davis-Smith 2007 (** **18** **)**	
Sample	N = 10 (7 women, 3 men) aged ≥18 years (mean and SD note reported) with prediabetes (FG 100–125mg/dl).
Theory	None noted
Study outcome(s)	Attendance, changes in FG, weight, BMI
Intervention	Duration: 6 weeks. Location: church. Design: 6-session group program (nutrition, PA, behavior change) derived from 16-session intensive lifestyle arm of NIH DPP. Education: diet and PA. Follow-up: 6 weeks, 6th month, 12th month.
Attendance and retention	Attendance: 78% overall sessions and participants. Retention: 90%.
Clinical outcomes and results	Weight: 4 kg loss. BMI: 1.7 decrease. FG: 7 mg/dL decrease. SBP: 3 mm Hg decrease. DBP: 5 mm Hg decrease. Results significant compared with baseline (*P* < .05) post-intervention and were maintained at 6th and 12th months.
**Wilson et al, 2005 (** **23** **)**	
Sample	N = 22 breast cancer survivors, women. Mean age, 55.0 years; SD, not reported.
Theory	Health behavior theory, SCT.
Study outcome(s)	Weight, BP, FG
Intervention	Duration: 2 months. Location: church. Design: individual; 16 sessions on exercise behaviors. Education: diet and PA. Follow-up: 3rd month.
Attendance and retention	Attendance: not reported. Retention: 65%.
Clinical outcomes and results	BMI: 0.38 decrease (*P* < .01). Weight: 0.9 kg decrease (*P* < .01). Waist circumference: 4.6 cm decrease (*P* = .04). SBP: 10.1 mm Hg decrease (*P* < .001). DBP: 6.2 mm Hg decrease (*P* < .01). Results post-intervention compared with baseline.

Abbreviations: BIA, bioelectrical impedance analysis; BMI, body mass index; BP, blood pressure; DBP, diastolic blood pressure; DXA, dual energy X-ray absorptiometry; FG, fasting plasma glucose; HbA1c, hemoglobin A1C; HDL, high-density lipoprotein; HOMA-IR, homeostasis model assessment-estimated insulin resistance; NIH DPP, National Institute of Health Diabetes Prevention Program; LDL, low-density lipoprotein; PA, physical activity; SBP, systolic blood pressure.

a References refer to the same study. Intervention results were published separately from attendance rates, retention rates, and strategies.

b References refer to the same study. Data were divided by physical activity- and diet-related content and were published separately.

### Clinical outcomes

Overall, the nutritional and physical activity interventions we reviewed reduced risk for chronic diseases by succeeding in improving clinically relevant outcome measures, including weight loss (13–19,22–24,30,32) and decreases in waist circumference (14,17,29), SBP (14,16,18,23–26,28), DBP (14,16,18,23–25), fasting plasma glucose (18,25), body fat percentage (14,16,19,23), hemoglobin A1c (16,31), and blood lipids (high-density lipoprotein [HDL], low-density lipoprotein [LDL], total cholesterol, and triglycerides) (14,24). Only 1 study, which was published in 2 separate articles as dietary and physical activity outcomes (20,21), was unable to produce significant results in 2 clinical outcomes: reducing BMI and waist circumference. Among the most commonly reported outcome measures (weight, BMI, SBP, and DBP), mean weight loss was 6.4 kg (range, 0.5–32.6 kg) (13–19,22–25,28,30), BMI decrease was 1.0 kg/m2 (range, 0–1.7 kg/m^2^) (15,17–19,22–25,28–30,32), SBP decrease was 6.7 mm Hg (range, 1.0–12.5 mm Hg) (12,14,16–19,23–26,28,32), and DBP decrease was 4.5 mm Hg (range, 1.0–10.2 mm Hg) (12,14,16–19,23–26,28,31,32). Weight loss seemed to vary widely; however, 1 study reported a weight loss of 32.6 kg resulting from a prescribed 1,000 kilocalorie diet (24); the next highest weight loss was 14.5 kg (22).

The improvements in serum biochemical markers, including HDL, LDL, and some other health indicators (eg, triglycerides, blood glucose levels) were measured and observed in just a few of the studies (17,19,32). Project Joy (17), a, church-group–based healthy-lifestyle intervention, reported significant decreases in LDL cholesterol in the intervention group and no changes in the self-help control group. However, a pilot, church-based, weight-loss program for African American adults using church members as health educators observed no significant changes in HDL, LDL, triglyceride, or blood glucose levels (19). The same was true for another study conducted in rural primary care health centers where the percentage of African American participants at each center ranged from 73.2% to 89.4% (32).

### Study setting and recruitment strategies

The majority of the 19 studies we reviewed were conducted in churches (n = 5), clinical or community health centers (n = 5), or other community locations (ie, community centers or local gyms) (n = 4). Remaining intervention locations were home-based (n = 1), universities (n = 2), a residence (n = 1), and a hospital (n = 1).

Recruitment methods varied depending on the intervention setting. Researchers conducting faith-based interventions (17–29,) generally announced the program in church bulletins, in the upcoming events section of church newsletters, at church services on Sundays, or at other church group meetings. They also relied on word of mouth and community contacts or delegated church pastors to make announcements about the program. Researchers conducting community and clinic-based interventions recruited participants via mailing lists (22), physician referrals (13,16,20,21,27), staff presentations (20,21), social networking and word of mouth (20,21), and targeted mass media (20,21). In an individual-based study (27), newspaper advertisements, flyers, and referrals from friends and coworkers were reported to be more effective recruitment methods (accounting for 73.0% of responses) than recruiting via blood pressure screenings, physician referrals, and Internet advertisements. Similarly, other researchers (20,21) found it easier to use personalized methods (ie, word of mouth or social networking) to recruit African American women with high BMI than African American women with low BMI (*P* = .01). Generally, 2 weeks to a month were spent recruiting participants before the start of the study. However, Staffileno and Coke (27) reported a recruitment period of 19 months because of the greater specificity of their desired population.

### Attendance and retention rates

Attendance rates at the 10 intervention programs that reported attendance varied from 33.0% to 95.0% (15,17,18,20–22,24,29–31). Various methods were reported for calculating attendance rates. One study (28) reported 85.0% and another (22) reported 55% of participants attended 75.0% of the sessions. One study reported an attendance rate of 95.0% for all 8 sessions, but that figure was based on an attendance requirement of only “at least one session” (20,21). The mean attendance rate of intervention programs that defined attendance as “participants who attended all sessions” (15,17,18,24,29–31) was 58.0%. Retention rates tended to be much higher (mean, 80.0%; range, 43.0%–96.0%) than attendance rates (mean, 58.0%; range, 33.6%–79.0%), suggesting it is more difficult to promote consistent participation in a program (16,18–22,25,26). Retention rates were also more commonly reported than attendance rates. Financial or other incentives were helpful for retention of participants; 1 study with a retention rate of 90.0% paid each participant $100 (19).

### Sustainability of outcomes

Although interventions show evidence of short-term (range, 2–6 months) improvements in diet and exercise habits, there was limited follow-up to prove that these changes were long-term and that they continued beyond the intervention. Of 6 studies that followed up with participants beyond the intervention, 4 studies (13,14,18,25) demonstrated sustainability of significant intervention-provoked outcomes at 12 months; others (20,21,23) did not. West et al (13) also observed that African American women had gained more weight than had whites, African American men, or Hispanics at a 30-month follow-up in a diabetes prevention program that used one-on-one counseling in a clinical center.

### Incorporation of behavior theory and randomization into study design

Of the studies reviewed, 7 (12,15,16,20–23,30) reported incorporating social cognitive theory as the theoretical framework for the intervention, and one (17) used social learning theory. Four studies combined social cognitive theory with a socioecological model (20,21), health belief model (22,23), transtheoretical model (12,15), and a community-based participatory research model (12). Eleven studies (13,14,18,24–26,29,31,32) did not report using a theory-based intervention design. Only 6 studies (15,18,22,23,25) did not report using a randomized clinical trial design.

### Educational interventions description

All of the 19 educational interventions reviewed (12–32) reported implementing physical activity education components, including goals for, benefits of, and strategies for increasing daily physical activity. A majority (78.9%, n =15) (12–20,22,24,25,30–32) of the 19 studies also implemented a dietary education component, including topics such as calorie reduction for weight loss, dietary sodium and fat reduction strategies, portion sizes, food group topics, and strategies for healthy eating while dining out and during the holidays. Four studies (13,18,25,32) followed the National Institutes of Health Diabetes Prevention Program (NIH DPP) curriculum for both diet and physical activity topics, whereas 1 study (12) used DASH (Dietary Approaches to Stop Hypertension) diet principles to guide nutrition education. Interventions (23,26,28,29) that focused only on education about physical activity for African American women were not significantly better at reducing weight, SBP, or DBP than were interventions (12–20,22,24,25,30–32) that focused on education about both physical activity and diet.

## Discussion

Because it has been confirmed that African Americans have an increased burden of chronic disease risk that is exacerbated by unhealthy diets and limited physical activity (34,35), interventions for diet and physical activity behavior modification may alleviate this burden. Several studies published since 2000 were available for review, and based on the current literature, diet and physical activity interventions targeting African Americans need to be refined. This review evaluated the most current literature on diet and physical activity interventions among African Americans, and we emphasize that clinical progress was a major consideration in our review. The evidence shows that nutrition and physical activity lifestyle modifications alone can significantly improve predictors of various clinical outcomes in African Americans (13–21,23–28), but more research is needed to substantiate the clinical significance of the outcomes.

In a more recent, similar review, Pezmeki and Jennings (10) examined physical activity outcomes without evaluating the effect of the behavior change on various health outcomes. Seven interventions (17,19–21,23,26,27,30) included in our analysis overlapped with the previous review. Because the review by Pezmeki and Jennings (10) aimed to examine physical activity outcomes exclusively, we saw a need to fill a gap in the literature by presenting a comprehensive examination of studies that promoted diet and physical activity behavior change to improve health outcomes, including major clinical indicators.

Although all 19 of the studies we reviewed demonstrated significance in various clinical outcomes, the most frequently reported variables were weight, BMI, SBP, and DBP. It was apparent that the African American participants in populations targeted by selected interventions were obese and prehypertensive. Mean decreases in SBP and DBP were sufficient to decrease blood pressure but not the number of people diagnosed with prehypertension (16–19,23–28,32). Although BMI remained in the obese range for participants in all 19 studies, mean weight loss reported amounted to approximately 3.0% of initial body-weight (13,15–19,22–25,28,30,32). Unfortunately, we did not find enough evidence to comment on the effect of diet and physical activity interventions on other clinical outcomes, including lipid and blood glucose levels.

Our findings are consistent with published research results that support using community-based settings for implementing interventions to improve diet and physical activity among African Americans. Our findings also indicate that churches may be useful in reaching communities beyond their congregations for study participation; several studies successfully recruited and reported changes among participants who did not attend church at the study’s location (18,23,29,31,32). In addition, we found that some of the highest participant retention rates were among programs conducted in churches (18,19).

Eleven studies (13,14,18,19,24–26,28,29,31,32) did not report using behavior theory in their intervention design, and this omission may indicate a weakness in the analyzed results of those studies. Unfortunately, compared with a past review of physical activity interventions among African Americans little progress has been made in increasing the number of studies that use health behavior theory in intervention program design (6). Research designed with a theoretical foundation better predicts and validates whether lifestyle interventions will promote changes in health behaviors while also providing a means to evaluate change processes in health behavior. Only about one-third of health-behavior–change studies conducted from 2000 through 2005 and reviewed by Painter et al (36) reported using behavior theory in intervention design. Our results indicate a slightly higher proportion; 42.0% of studies used behavior theory models. Furthermore, of the 8 studies (12,15–17,20–23,30) that used a behavior theory, 7 reported significant results (12,15–17,22,23,30), whereas the remainder of studies (13,14,18,19,24–26,29,31,32) all reported significant results. It is possible that studies examined may lack explicit reporting of the use of behavior theories. We suggest that common health behavior theories be incorporated into intervention design, applied more thoroughly in research beyond mere consideration of their definition, tested in African Americans, and modified as necessary to better suit the population (36).

This review found no differences in select clinical outcomes (eg, weight, SBP, and DBP) between programs that offered physical activity education and those that offered physical activity plus diet education, although few physical activity-education only interventions were available for comparison. One study included in this review (14) was noteworthy in that it found no differences in body composition (weight, waist circumference, and BMI) at a 6-month interval between participants who received a diet education intervention and those who received a diet plus physical activity intervention. This lack of difference suggests that both diet and physical activity should be considered major components of a lifestyle behavior change intervention until further evidence is available and examined. Other comparison issues noted were the lack of homogeneity among the education tools used in interventions and our inability to determine which specific education tools were used. Without this information, it is hard to generalize, repeat, and retest successful education programs as the NIH DPP has done (13,18,25,32).

Compared with programs aimed at improving physical activity and diet in predominately white populations, mean attendance and retention rates at programs for African Americans were lower. The mean attendance rate for studies with predominantly white participants was 95.0% (37–39) compared with 58.0% for studies in our review with predominantly African American participants (12,15,17,18,22,24,31). In addition, studies with mostly white participants reported a mean retention rate of 87.0% (37,38) compared with a mean of 80.0% reported for studies with mostly African American participants (13–32). Several studies (39–43) identified effective retention practices for African American research participants, but because culture plays a central role in behavior choices, generic health messages may conflict with the cultural beliefs of minority populations (44). The increasing prevalence of coronary vascular disease among African Americans illustrates the need for culturally relevant associations between food and health, including the use of culturally tailored intervention components, such as lifestyle-change education to lower coronary vascular disease risk by modifying, not eliminating, cultural foods to improve diet and by suggesting culturally acceptable and attractive modes of physical activity. It is still necessary to improve both African American attendance and retention rates for health interventions. However, more than half of the studies in the current investigation (n = 10) did not report an attendance rate (13,14,16,19–21,23–26,32), thereby making attendance unavailable for evaluation. Not reporting attendance is notable because program attendance is imperative for participants to acquire the knowledge and skills needed to modify health behaviors (45).

Our study had limitations. Our review was only as good as the available studies analyzed. Not all included studies used randomization for participant allocation, and some of the results may show allocation bias. Another limitation is publication bias. Many relevant studies that were unpublished because of nonsignificant results would have been included in this review if published. Other analyzed studies did not use control groups for comparison, which may have introduced some experimenter bias for the overall conclusions.

This review provides researchers with current information on intervention program effectiveness and provides policy makers and health care practitioners with evidence about whether nutrition education and physical activity promotion interventions are valuable methods for improving the health of African Americans. The authors identified from the reviewed literature a common protocol for implementation of lifestyle interventions to prevent chronic disease in African American adults (Appendix). To our knowledge, there is no previously published analysis that encompasses all variables considered in this systematic review.

Nutrition and physical activity interventions promote positive changes in the health behaviors of adult African Americans by providing them with knowledge and resources about disease prevention. The reviewed studies have shown that these health interventions had a positive effect on the participants’ dietary choices and physical activity habits, which translated to clinically relevant outcomes in communities, churches, and health clinics. Using health education and interventions designed to teach African American adults about healthy choices (eg, proper nutrition and adequate exercise) empowers them to make necessary lifestyle changes. Thus, potential exists for reducing preventable risks for diseases and comorbidities while positively affecting the health of the community. Future interventions should incorporate theoretical models appropriate for health-related issues and randomized controlled trial design into the basis of their programming, because this gives credence to evidence-based research.

The current literature suggests that more research is needed to determine the cost and sustainability of lifestyle intervention programs. We noticed in our review that the costs of designing and implementing interventions were rarely published, offering no guidance as to what the typical costs are of interventions. Additionally, a cost-benefit analysis would facilitate the awareness and spread of information about public policy and funding allocation. Sustainability of improved diet and physical activity behavior is also rarely noted. Programs are implemented and results are documented, but whether targeted populations are able to further or maintain progress after the cessation of the intervention is still in question.

Further research is required to substantiate the link between the intervention-induced diet and physical activity changes and related disease-risk biochemical markers. Such data could be documented on a clinical level, and the information on population disease-risk characteristics could become available. This type of data also adds to the potency of the argument for funding public education programs. Although many interventions have proven successful for African American populations, filling the information gaps will promote and substantiate further progress of intervention research and a subsequent improvement in overall health of African Americans and a reduction in population health disparities.

## Appendix. Protocol Based on Published Research Findings About Implementing a Lifestyle Intervention Program for African American Adults

1. Identify target population.

2. Identify appropriate location for intervention.

3. Develop relationships with appropriate leaders.

4. Recruit members of the community, staff of a clinical center, university professors/students, etc., to represent the implementation team.

5. Utilize focus groups to determine the needs, values, health beliefs of intervention population.

6. Develop appropriate agenda/schedule for all phases of the intervention, including introduction of the project, baseline (before measurements) and follow-up measurements, and programming.

7. Publicize brand of the project, project goals, program dates, and recruitment criteria.

8. Begin recruitment with signed consent forms and appropriate privacy protection procedures.

9. Implement the program with the goals and interests of the target population in mind.

10. Support participation via phone calls, letters, e-mails, and reminders of sessions.

11. Increase interest by creating newsletters about study milestones and some individual or group progress.

12. If applicable, create Web page for the study with accompanying accesses to Twitter, Facebook, and such.
